# Comparison of liver stereotactic body radiotherapy plans based on free breathing and averaged CTs after trans‐arterial chemoembolization using lipiodol

**DOI:** 10.1002/acm2.70271

**Published:** 2025-09-29

**Authors:** Sei‐Kwon Kang, Jai‐Woong Yoon, Me Young Kim, Soah Park, Kwang‐Ho Cheong, Taeryool Koo, Tae Jin Han

**Affiliations:** ^1^ Department of Radiation Oncology Kangdong Sacred Heart Hospital Hallym University College of Medicine Seoul South Korea; ^2^ Department of Radiation Oncology Dongtan Sacred Heart Hospital Hwaseong South Korea; ^3^ Department of Radiation Oncology Chuncheon Sacred Heart Hospital Hallym University College of Medicine Chuncheon Gangwon‐do South Korea; ^4^ Department of Radiation Oncology Kangnam Sacred Heart Hospital Hallym University College of Medicine Seoul South Korea; ^5^ Department of Radiation Oncology Hallym University Sacred Heart Hospital Hallym University College of Medicine Anyang South Korea

**Keywords:** 4D cumulative dose, average intensity projection, free breathing, lipiodol, liver stereotactic body radiotherapy

## Abstract

**Purpose:**

To identify which CT set is more suitable for predicting the four‐dimensional (4D) cumulative dose distribution in liver stereotactic body radiotherapy (SBRT) with lipiodol retention.

**Methods:**

For 15 patients, who underwent liver SBRT after trans‐arterial chemoembolization (TACE), volumetric modulated arc therapy (VMAT) plans were retrospectively generated on free breathing (FB) and average intensity projection (AVG) CT sets using the x‐ray Voxel Monte Carlo (XVMC) algorithm. The three‐dimensional (3D) FB and AVG plans were compared with corresponding 4D plans, which were created by deformable registration using 10‐phase plans. For both internal target volume (ITV) and planning target volume (PTV), the volume covered by the prescription dose of 36 Gy (V36) and the dose covering 95% of the volume (D95) were evaluated. The relative (3D/4D) normal liver volume >19.2 Gy and rib volume  28.8 Gy were also compared for FB and AVG plans.

**Results:**

The mean values of the relative electron densities inside the PTV for FB and AVG were slightly different (1.050 for FB vs. 1.039 for AVG). For both ITV and PTV, V36 and D95 showed no statistically significant difference between FB and AVG. Also, the relative rib volumes >28.8 Gy and the normal liver volumes >19.2 Gy were not statistically different between FB and AVG plans.

**Conclusion:**

For liver SBRT with lipiodol retention, 3D plans based on FB and AVG images were virtually equivalent in producing the 4D cumulative doses under the accuracy of the XVMC algorithm.

## 1 INTRODUCTION

Stereotactic body radiotherapy (SBRT) for liver cancer is commonly practiced after trans‐arterial chemoembolization (TACE) using lipiodol as an embolization agent.^1^, ^2^ Lipiodol is an iodine‐based high‐density material and has shown potential as a tumor surrogate for target localization in radiotherapy due to its high contrast in the CT images.^3^ The degree of lipiodol retention before radiotherapy has also been suggested as a prognostic factor for liver patients.^4^

Two issues related to lipiodol in liver SBRT need to be noted: dose enhancement by lipiodol and respiratory‐induced variation in the location and density of the tumor containing the lipiodol. Dose enhancement by high‐density materials in radiation treatment has been previously investigated.^5^, ^6^, ^7^ For example, Zhang et al. studied a gadolinium‐based contrast agent in megavoltage photon beams using Monte Carlo (MC) simulation. The dose enhancement depended on the beam spectrum and agent concentration. For the Ir‐192 source used in high dose rate brachytherapy, about a fourfold enhancement was observed compared with that of a conventional 6 MV beam.^6^ The dose enhancement by lipiodol was studied by Kawakara et al.^8^ According to the full MC simulations using the BEAMnrc/DOSXYZnrc for phantom slabs and a clinical case, the average dose in the lipiodol uptake region increased by 8.1 and 6.0%, respectively, compared with those calculated by the commercial dose calculation algorithm AcurosXB (Varian Medical Systems, Palo Alto, USA).

The other problem is the tumor's change in location and density due to respiratory motion, since lipiodol is a high‐density material inside tissue. This situation is similar to a lung tumor moving during breathing within the surrounding lung tissue. Various approaches to lung tumor motion for accurate cumulative dose distribution have been reported.^9^, ^10^, ^11^ According to Tian et al., in a lung SBRT plan study, plans based on free breathing (FB) and average intensity projection (AVG) CT sets produced similar dosimetric characteristics with clinically acceptable differences for cumulative lung doses.^9^ Clinical equivalence of FB and AVG images for lung dose calculation was also reported by Zvolanek et al., using AXB (Eclipse, Varian) and MC calculations.^10^ In addition to deciding which CT scan is more adequate for accurate dose evaluation, density override of lower lung tissue was studied to compensate for probable dose deficiency in target coverage and to spare surrounding lung tissue from over‐irradiation.^11^

In this study, based on FB and AVG CT sets with lipiodol retention, we compared 3D VMAT plans with their corresponding cumulative 4D plans to determine which CT set was more reliable for predicting respiration‐compensated cumulative dose in liver SBRT.

## 2 METHODS

Retrospectively, 15 patients who were treated with liver SBRT after TACE were selected based on visual inspection of CT images showing lipiodol retention with contrast similar to that of bone. Patients were immobilized using Elekta BodyFix and CT scanned for conventional three‐dimensional (3D) images as well as 4D CT sets with 10 respiratory phases. The Canon Aquilion CT (Canon Medical Systems, Ohtawara, Japan) and Abches (APEX Medical Inc., Tokyo, Japan) for respiration monitoring were used.

After contouring the gross tumor volume (GTV), including lipiodol on the 3D images, an internal target volume (ITV) was generated by adjusting the GTV to compensate for tumor motion on each phase image and was further expanded isotropically by 5 mm to create the planning target volume (PTV) with a setup margin. VMAT plans were created on the FB CT sets using 10 MV of an Elekta VersaHD linear accelerator and the Monaco planning system (version 5.51), with a prescription dose of 36 Gy delivered in four fractions, covering 95% of the PTV. The dose calculation algorithm used was the fast MC method called x‐ray Voxel Monte Carlo (XVMC), and the dose calculation grid was set to 2.0 mm.

To assess the 4D cumulative dose, the plan was copied to each phase of the 4D CTs, and doses were recalculated. The doses were accumulated on the FB CT as a reference CT using MIM's deformable registration software (MIM Maestro v7.0, MIM Software Inc., USA), applying equal weighting of one tenth for each phase, assuming that respiratory motion was temporally uniform. In this study, 4D plans refer to the reference CT sets with cumulative dose distributions from all respiratory phase plans. The procedures to create the 3D and 4D plans using the FB images were repeated on the AVG CT images. The AVG image set was generated by averaging the intensities of all phase CTs. After copying all contours for normal organs and PTV from the 3D FB plan to the AVG images with no modifications, the AVG plan was generated through parameter optimization. All dose distributions were also accumulated on the FB images for comparison. During the acquisition of the 4D cumulative dose distribution for each patient, the default registration options were used for both FB and AVG plans to eliminate the influence of potential registration inaccuracies.

For both ITV and PTV in the 4D plans, the volume covered with the prescription dose of 36 Gy (V36) and the dose covering 95% of the volume (D95) were evaluated. The ratios of the 3D to 4D volumes for normal liver volume (excluding the PTV) receiving >19.2 Gy and rib volume receiving >28.8 Gy were compared between the FB and AVG plans.^12^ Statistical analysis was performed using the Wilcoxon signed‐rank test, with a *p*‐value <0.05 considered statistically significant.

## 3 RESULTS

Some of the target characteristics and statistical results for 15 patients are listed in Table 1. The motion range of the targets, simply measured in the superior–inferior (SI) direction, was 0.6–2.1 cm (mean 1.16 cm).

**TABLE 1:** Target information and selected evaluation results of 15 patients.

	Mean (range)		
	FB	AVG	*p*‐value
PTV volume (cm^3^)	40.2 (9.7−132.3)		
Motion (SI: cm)	1.16 (0.6−2.1)		
lipiodol electron density	1.209 (1.109−1.313)		
PTV electron density	1.050 (0.922−1.098)	1.039 (0.894−1.080)	0.029
4D ITV 95% dose (Gy)	36.6 (35.0−37.5)	37.0 (35.1−39.1)	0.251
4D ITV 36 Gy vol (%)	97.4 (90.1−100)	97.5 (90.7−100)	0.738
4D PTV 95% dose (Gy)	32.2 (27.5−35.5)	32.1 (26.7−36.5)	0.247
4D PTV 36 Gy vol (%)	80.5 (59.1−91.9)	79.8 (56.4−97.7)	0.169
Normal liver vol > 19.2 Gy (%)^a^	91.9 (61.8−125.4)	92.9 (62.1−127.4)	0.095
Rib vol > 28.8 Gy (%)^a^	100.7 (71.7−130.1)	94.1 (65.5−108.3)	0.813

Abbreviation: SI, superior–inferior.

^a^ Relative value (3D/4D).

### 3.1 Relative electron density

**FIGURE 1:**
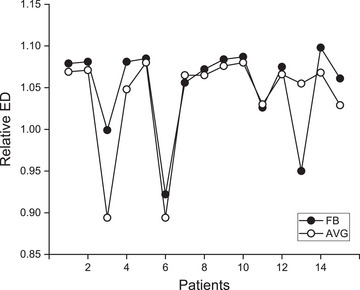
Relative electron density values inside the PTVs for FB and AVG images.

Relative electron densities for each PTV of the FB and AVG images are shown in Figure 1. The range of density values for FB and AVG sets was significantly different (*p* = 0.029), with 0.922–1.098 (mean: 1.050) for FB and 0.894–1.080 (mean: 1.039) for AVG images, respectively. As expected, the mean density for the PTV of the AVG set was smaller than that of the FB set due to smearing caused by organ motion. The averages of the standard deviations (1 sigma) of electron densities for each PTV were 0.114 and 0.099 for FB and AVG images, reflecting slightly more uniform electron densities inside the PTV of the AVG set, though the difference was not significant (*p* = 0.246).

**FIGURE 2:**
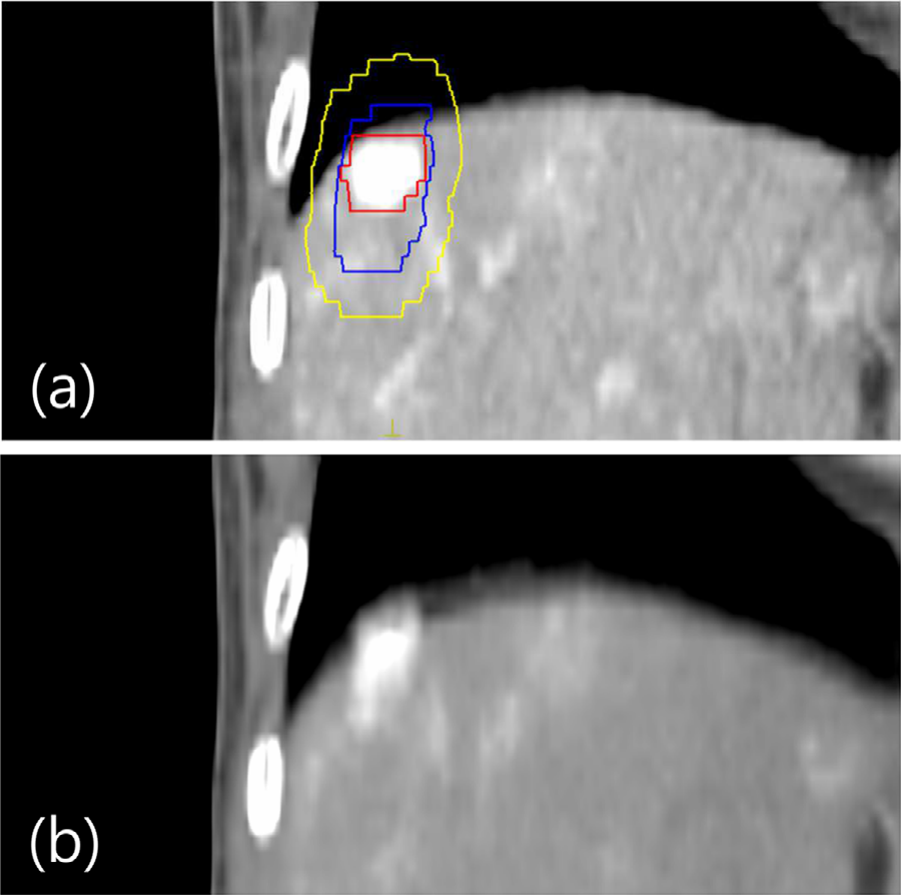
Coronal view example of Patient #6. (a) FB and (b) AVG images. Contours for the GTV (red) containing lipiodol, ITV (blue), and PTV (yellow) are overlaid. Organ boundaries appear blurred on the AVG image.

Patients #3 and #6 showed markedly lower values; for the FB image, the electron densities were 0.999 (SD = 0.286) for Patient #3 and 0.922 (SD = 0.31) for Patient #6. For the AVG images, values were 0.894 (SD = 0.341) for Patient #3 and 0.894 (SD = 0.262) for Patient #6. For these two patients, their GTVs were located close to the lung diaphragm. Figure 2 shows an example of coronal images for Patient #6, where the ITV and PTV extend into the lung region. Electron density values for each region were 0.324 (SD = 0.088) for lung, 1.298 (SD = 0.006) for lipiodol, and 1.081 (SD = 0.003) for the tissue inside the PTV. When the treatment volume is near the diaphragm, the target may be displaced into regions with varying density environments.

### 3.2 Volume coverage and dose of 4D ITV and PTV

**FIGURE 3:**
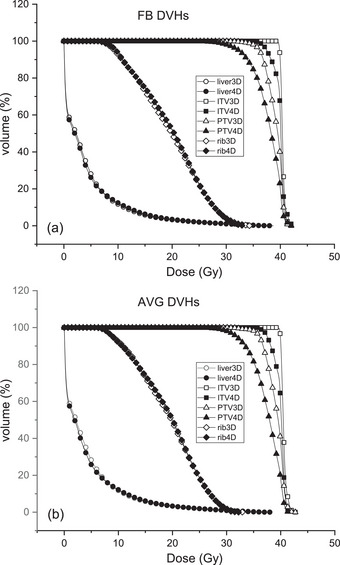
Example of 3D and 4D DVHs for Patient #6. (a) FB and (b) AVG plan. DVHs for the liver represent the normal liver tissue.

Figure 3 shows examples of 3D and 4D dose–volume histograms (DVHs) of the FB and AVG plans for Patient #6. For both plans, the respiration‐corrected 4D DVHs of ITV and PTV showed decreased dose coverage. The DVHs for the normal liver and rib for the 3D and 4D plans were very similar for this patient. The degree of difference in dose distributions between 3D and 4D was patient‐dependent.

As shown in Table 1, doses to 95% (D95) of the 4D ITV were, on average, 36.6 Gy (range: 35.0–37.5 Gy) and 37.0 Gy (35.1–39.1 Gy), and the volume receiving more than the prescribed dose of 36 Gy (V36) was 97.4% (90.1%–100%) and 97.5% (90.7%–100%) for FB and AVG plans, respectively, with no statistical differences.

**FIGURE 4:**
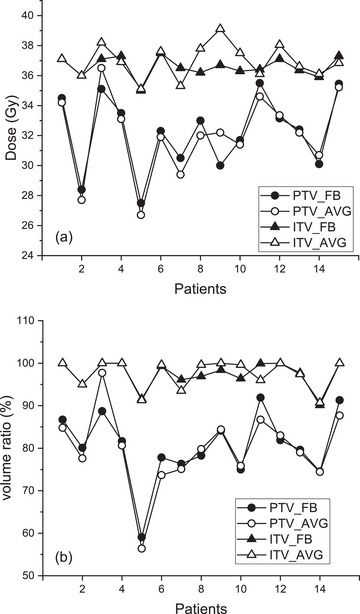
Cumulative 4D analysis for FB and AVG. (a) Dose covering 95% of ITV and PTV (D95), and (b) volumes covered by the prescribed dose of 36 Gy (V36).

For the 4D PTV, D95 and V36 showed no significant differences between FB and AVG plans and were largely patient‐dependent (Figure 4). The D95 doses ranged from 27.5 to 35.5 Gy (mean 32.2 Gy) for FB and from 26.7 to 36.5 Gy (mean 32.1 Gy) for AVG plans. For V36, values ranged from 59.1 to 91.9% (mean 80.5%) for FB and 56.4 to 97.7% (mean 79.8%) for AVG. Considering that both 3D plans for FB and AVG were created to irradiate 95% of the PTV by 36 Gy, a patient‐dependent large variation of the respiration‐compensated cumulative D95 and V36 was notable.

### 3.3 Normal liver and rib

**FIGURE 5:**
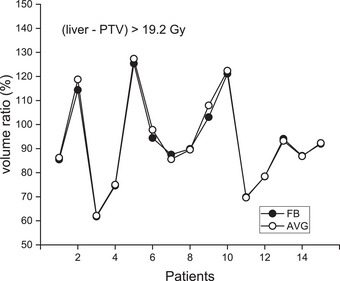
Relative normal liver volumes (3D/4D) receiving more than 19.2 Gy (liver minus PTV) for FB and AVG plans.

Relative (3D/4D) normal liver volumes receiving doses over 19.2 Gy, excluding the PTV, were evaluated and are shown in Figure 5 for FB and AVG plans.^12^ The volume ratios for FB and AVG plans were not statistically different (*p* = 0.095), ranging from 61.8 to 125.4% (mean 91.9%) for FB and 62.1 to 127.4% (mean 92.9%) for AVG plans. Therefore, in predicting 4D accumulated normal liver volumes (>19.2 Gy), the 3D FB and AVG plans were equivalent. The normal liver volume ratio was largely patient‐dependent. For Patient #3, both FB and AVG 3D plans predicted the normal liver volume >19.2 Gy to be 61.8 and 62.1% of their corresponding 4D volumes, respectively. In contrast, for Patient #5, these values were 125.4 (FB) and 127.4% (AVG) of the 4D values.

**FIGURE 6:**
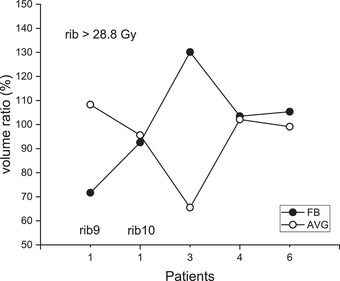
Relative rib volumes (3D/4D) receiving doses greater than 28.8 Gy. Two cases correspond to Patient #1.

A similar analysis was performed for the ribs close to the target to evaluate the probable rib fracture. Five cases among 15 patients (including two cases from Patient #1) showed ribs very close to the treatment target. Rib volumes irradiated with more than 28.8 Gy were obtained for both FB and AVG 3D and 4D plans, and the ratio of 3D to 4D values was evaluated.^12^ The volume ratio ranged from 71.7 to 130.1% (mean 100.7%) for FB and 65.5 to 108.3% (mean 94.1%) for AVG plans. The number of samples was too small to draw definitive conclusions. However, with this limited data, FB and AVG 3D plans showed no difference in evaluating cumulative rib dose (*p* = 0.813). Individual data are displayed in Figure 6. For Patient #1, the ninth rib (rib9) and tenth rib (rib10) were included in the assessment. While these two ribs belonged to the same patient, FB and AVG plans produced underestimation (71.7%) and overestimation (108.3%) for rib9, respectively. For rib10, similar estimates of 92.6% (FB) and 95.6% (AVG) were obtained. For Patient #3, FB overestimated the rib dose (130.1%), while AVG underestimated it (65.5%).

## 4 DISCUSSION

Recently, Kang et al. noted that high‐density lipiodol inside the liver tumor resembles a solid tumor inside lung tissue under breathing motion.^13^ Respiratory motion shifts the tumor into a new environment where photon fluence differs from the initially optimized one in VMAT planning. This raises concerns about proper tumor irradiation, since the moving target could consist of tumor tissue, lipiodol with higher density, and lung tissue with lower density if the tumor is near the diaphragm. They studied the effect of lipiodol on target coverage through 4D evaluation by considering tumor tissue and lipiodol regions separately. Once the GTV was covered with the prescribed dose, the tumor tissue inside the GTV received a higher dose according to their results.

The aim of this work was to explore which CT set—FB or AVG—was more reliable to predict the cumulative dose for a moving liver target, specifically when the target contains the high‐density material lipiodol. To this end, 3D plans based on FB and AVG images were compared with their corresponding 4D plans.

### 4.1 FB versus AVG plans for cumulative dose distribution

In our study, the electron density of AVG images was slightly more uniform due to image smearing. This reduced inhomogeneity was expected to cause photon fluences to be less sensitive to variations in each phase image. From lung SBRT plans calculated using XVMC, Zvolanek et al. found no statistical differences between FB and AVG for dosimetric parameters of target coverage and reported that FB and AVG CTs were equivalent for dose calculation.^10^ Since the density difference between lipiodol and liver tissue is smaller than that between lung and solid tumor, the equivalency of FB and AVG in our study is not surprising. Lipiodol was unlikely to affect the dose distribution in liver SBRT.

For relative (3D/4D) dose–volume parameters (D95 and V36) of ITV and PTV, FB and AVG plans also showed no statistically significant differences. Hence, in predicting 4D dose and volume metrics for liver SBRT after TACE, FB and AVG plans were considered equivalent. The differences between 3D and 4D values were largely patient‐dependent.

The large fractional dose in liver SBRT raises concerns about doses to normal organs. In our study, normal liver volumes receiving a dose greater than 19.2 Gy were evaluated as an indicator of probable radiation‐induced liver disease (RILD).^12^ When the ratios of that volume in 3D plans (FB, AVG) to the corresponding 4D volumes were compared, differences were small; 91.9 versus 92.9% for FB and AVG plans, respectively (*p* = 0.095). Both FB and AVG plans reproduced more than 95% of the normal liver volumes obtained from 4D accumulations.

The 3D‐to‐4D volume ratio of ribs receiving more than 28.8 Gy was also evaluated for five cases. Although the delineated rib volumes were small and deformable registration may include uncertainties, the volume ratio of 3D relative to 4D was highly patient‐dependent and showed no significant difference between FB and AVG plans.

In summary, for liver SBRT with the high contrast of lipiodol, FB and AVG images were equivalent for estimating dosimetric quantities—including dose coverage of ITV and PTV, normal liver volumes, and dose to ribs under the accuracy of the XVMC dose calculation algorithm.

### 4.2 Reduction of the 4D coverage

The reduction in 4D dosimetric parameters (V36 and D95) for the PTV was notable. Mean values for FB and AVG plans showed that approximately 98% of the ITV volume was irradiated by the prescribed dose (36 Gy), while 95% of the ITV volume received 37 Gy. In contrast, the PTV coverage was only about 80%, with D95 around 32 Gy. Insufficient 4D coverage of the moving target has been reported by several researchers.^14^, ^15^, ^16^ For example, Yeo et al. found that AVG‐based 3D plans overestimated target dose by up to 8% and underestimated normal liver dose compared to accumulated 4D plans.^16^ Reduction of GTV coverage in 4D assessments has also been reported, with most discrepancies attributed to setup errors.^15^

Intuitively, the reduction in 4D dosimeric parameters is inevitable for moving targets. During the registration process for accumulated 4D doses, the peripheral region outside the target—typically irradiated with much lower doses—could be translated and combined into the PTV in the reference plan. Therefore, the large drop in 4D coverage mainly arises from the target periphery lying on the steep gradient of the prescription dose.^17^ Another probable factor is the deformable registration. In our clinic, physicians create the ITV by manually adjusting target contours for each phase image, which does not guarantee the same ITV as formed by registration tools. If a 4D dose by deformable registration is used as ground truth, the target should also be created by the registration tool. However, this may not always be ideal. For lung cancer patients, Yakoumakis et al. contoured the GTV on the end‐exhale phase CT and generated GTVs for other phases using deformable registration; however, the ITV based on all phases showed lower coverage than the reference plan.^18^ The accuracy of liver deformable registration has been questioned due to the lack of anatomical landmarks.^19^ A registration accuracy of <1.5 mm has been reported for the tool (MIM) used in this study.^20^ In our cases, high‐contrast lipiodol could serve as a registration marker, improving accuracy. Nevertheless, respiratory phase images showed shape and density variation in the lipiodol region, making registration uncertain. Further investigation of deformable registration accuracy, both locally and globally, including patient‐specific evaluation, is warranted.^21^ Still, the equivalence of FB and AVG images for planning in our study was unaffected by registration accuracy since identical default registration parameters were used for both CT sets.

A practical approach to increase 4D target coverage is to add larger margins to the ITV. However, this increases the volume of normal tissue exposed to high doses, thereby raising RILD risk. For example, Xu et al. reported that while plans based on FB 3D CT showed large reductions in 4D cumulative PTV coverage, plans based on maximum intensity projection (MIP) images maintained nearly the same 4D coverage as 3D. However, normal liver volumes irradiated under MIP were about 1.5 times those in FB plans.^3^ In principle, tumor tracking may be the only viable option for treating moving targets, as it was planned without further increasing the irradiated volume of normal organs. To balance 4D target coverage and normal organ sparing, Starkschall et al. proposed a replanning approach with specific criteria for allowable dosimetric differences between 3D and 4D.^17^ For routine 4D evaluation, Yeo et al. recommended using at least three‐phase CT sets (two extreme phases and one mid‐phase) to reduce 4D planning workload instead of all respiratory phases.^16^ Each clinic needs to establish its own protocol to address the reduction in 4D dose–volume parameters.

### 4.3 Dose calculation for lipiodol retention

As discussed by Kawakara et al. regarding dose enhancement due to lipiodol, the commercial dose calculation algorithm AcurosXB (Varian Medical Systems, Palo Alto, USA) was not sufficient to predict this enhancement. This limitation was attributed to the lack of proper material information on iodine, a constituent of lipiodol, in the dose calculation algorithm.^8^ During dose calculation, the lipiodol region was treated as bone in terms of both density and material properties.

A similar limitation was applied to XVMC, which is a fast MC engine based on material information specific to the human body.^13^, ^22^ Therefore, the material properties of lipiodol were not considered in XVMC calculations, and dose enhancement effects due to lipiodol were not incorporated.^8^, ^13^, ^22^ Likewise, XVMC treats lipiodol as high‐density bone based on CT numbers without considering the chemical composition of lipiodol—unlike full MC engines such as BEAMnrc/DOSXYZnrc. Beyond proper material characterization, the spatial distribution of lipiodol must also be considered for accurate dose calculation. The irregular shape of highly concentrated lipiodol regions and their dispersion within the liver necessitate automatic identification of their location and intensity. As suggested by Kang et al., planning systems incorporating lipiodol information and spectral CT to identify lipiodol distribution would improve liver SBRT plan quality based on high‐contrast lipiodol CT images.^13^

The lack of material information for lipiodol in the dose calculation algorithm could locally affect the generation of photon fluence to irradiate the PTV in FB and AVG plans. This could, in turn, influence the subsequent 4D evaluation using each phase CT set. Therefore, it should be noted that the equivalence between FB and AVG plans for predicting cumulative 4D doses in this study is valid within the accuracy inherent to the XVMC fast MC dose calculation algorithm.

## 5 CONCLUSION

For liver SBRT planning on CT scans showing lipiodol retention, FB and AVG scans are interchangeable for predicting cumulative 4D dose distribution with respect to target coverage by the prescription dose, normal liver volumes, and rib dose sparing. It should be noted that the XVMC algorithm requires further improvement to accurately calculate doses in regions containing lipiodol by incorporating material‐specific information.

## AUTHOR CONTRIBUTIONS

*Study design*: Sei‐Kwon Kang, Soah Park, and Me Young Kim. *Literature research*: Sei‐Kwon Kang, Soah Park, and Me Young Kim. *Manuscript preparation*: Sei‐Kwon Kang, Soah Park, and Me Young Kim. *Planning and data collection*: Sei‐Kwon Kang, Soah Park, Kwang‐Ho Cheong, Jai‐Woong Yoon, and Me Young Kim. *Tumor delineation*: Taeryool Koo, Tae Jin Han, and Kwang‐Ho Cheong. *Plan analysis*: Taeryool Koo, Tae Jin Han, and Kwang‐Ho Cheong. All authors read and approved the manuscript.

## CONFLICT OF INTEREST STATEMENT

The authors declare no conflicts of interest.

## References

[1] Nezami N , Van Breugel JNN , Konstantinidis M , et al. Lipiodol deposition and washout in primary and metastatic liver tumors after chemoembolization. In Vivo. 2021;35(6):3261‐3270. 34697157 10.21873/invivo.12621 PMC8627740

[2] Yue J , Sun X , Cai J , et al. Lipiodol: a potential direct surrogate for cone‐beam computed tomography image guidance in radiotherapy of liver tumor. Int J Radiat Oncol Biol Phys. 2012;82(2):834‐841. 21377291 10.1016/j.ijrobp.2010.12.050

[3] Xu H , Gong G , Yin Y , Liu T . A preliminary investigation of re‐evaluating the irradiation dose in hepatocellular carcinoma radiotherapy applying 4D CT and deformable registration. J Appl Clin Med Phys. 2021;22(2):13‐20. 33452706 10.1002/acm2.13111 PMC7882094

[4] Yang P , Zeng ZC , Wang BL , et al. The degree of lipiodol accumulation can be an indicator of successful treatment for unresectable hepatocellular carcinoma (HCC) patients—in the case of transcatheter arterial chemoembolization (TACE) and external beam radiotherapy (EBRT). J Cancer. 2016;7(11):1413‐1420. 27471557 10.7150/jca.15405 PMC4964125

[5] Alkhatib A , Watanabe Y , Broadhurst JH . The local enhancement of radiation dose from photons of MeV energies obtained by introducing materials of high atomic number into the treatment region. Med Phys. 2009;36(8):3543‐3548. 19746788 10.1118/1.3168556

[6] Zhang DG , Feygelman V , Moros EG , Latifi K , Zhang GG . Monte Carlo study of radiation dose enhancement by gadolinium in megavoltage and high dose rate radiotherapy. PLoS One. 2014;9(10):e109389. 25275550 10.1371/journal.pone.0109389 PMC4183586

[7] Jackson N , Cecchi D , Beckham W , Chithrani DB . Application of high‐Z nanoparticles to enhance current radiotherapy treatment. Molecules. 2024;29(11):2438. 38893315 10.3390/molecules29112438 PMC11173748

[8] Kawahara D , Ozawa S , Saito A , et al. Dosimetric impact of Lipiodol in stereotactic body radiation therapy on liver after trans‐arterial chemoembolization. Med Phys. 2017;44(1):342‐348. 28102954 10.1002/mp.12028

[9] Tian Y , Wang Z , Ge H , et al. Dosimetric comparison of treatment plans based on free breathing, maximum, and average intensity projection CTs for lung cancer SBRT. Med Phys. 2012;39(5):2754‐2760. 22559646 10.1118/1.4705353

[10] Zvolanek K , Ma R , Zhou C , et al. Still equivalent for dose calculation in the Monte Carlo era? A comparison of free breathing and average intensity projection CT datasets for lung SBRT using three generations of dose calculation algorithms. Med Phys. 2017;44(5):1939‐1947. 28273341 10.1002/mp.12193

[11] Aarup LR , Nahum AE , Zacharatou C , et al. The effect of different lung densities on the accuracy of various radiotherapy dose calculation methods: implications for tumour coverage. Radiother Oncol. 2009;91(3):405‐414. 19297051 10.1016/j.radonc.2009.01.008

[12] Benedict SH , Yenice KM , Followill D , et al. Stereotactic body radiation therapy: the report of AAPM Task Group 101. Med Phys. 2010;37(8):4078‐4101. 20879569 10.1118/1.3438081

[13] Kang SK , Yoon JW , Kim MY , et al. Four‐dimensional dose evaluation for the liver stereotactic body radiotherapy with lipiodol retention after transcatheter arterial chemoembolization. J Radiat Res Appl Sci. 2024;17(1):100825.

[14] Ma C , Duan J , Yu S , Ma C . Dosimetric study of three‐dimensional static and dynamic SBRT radiotherapy for hepatocellular carcinoma based on 4DCT image deformable registration. J Appl Clin Med Phys. 2020;21(2):60‐66. 10.1002/acm2.12811 PMC7020978 31889422

[15] Velec M , Moseley JL , Craig T , Dawson LA , Brock KK . Accumulated dose in liver stereotactic body radiotherapy: positioning, breathing, and deformation effects. Int J Radiat Oncol Biol Phys. 2012;83(4):1132‐1140. 22208969 10.1016/j.ijrobp.2011.09.045 PMC3337347

[16] Yeo UA , Taylor ML , Supple JR , et al. Evaluation of dosimetric misrepresentations from 3D conventional planning of liver SBRT using 4D deformable dose integration. J Appl Clin Med Phys. 2014;15(6):4978. 25493523 10.1120/jacmp.v15i6.4978 PMC5711129

[17] Starkschall G , Britton K , McAleer MF , Jeter MD , Kaus MR , Bzdusek K , et al. Potential dosimetric benefits of four‐dimensional radiation treatment planning. Int J Radiat Oncol Biol Phys. 2009;73(5):1560‐1565. 19231098 10.1016/j.ijrobp.2008.12.024

[18] Yakoumakis N , Winey B , Killoran J , et al. Using four‐dimensional computed tomography images to optimize the internal target volume when using volume‐modulated arc therapy to treat moving targets. J Appl Clin Med Phys. 2012;13(6):3850. 23149778 10.1120/jacmp.v13i6.3850 PMC5718550

[19] Ehrbar S , Lang S , Stieb S , et al. Three‐dimensional versus four‐dimensional dose calculation for volumetric modulated arc therapy of hypofractionated treatments. Z Med Phys. 2016;26(1):45‐53. 26187810 10.1016/j.zemedi.2015.06.010

[20] Guy CL , Weiss E , Che S , Jan N , Zhao S , Rosu‐Bubulac M . Evaluation of image registration accuracy for tumor and organs at risk in the thorax for compliance with TG 132 recommendations. Adv Radiat Oncol. 2019;4(1):177‐185. 30706026 10.1016/j.adro.2018.08.023 PMC6349597

[21] Paganelli C , Meschini G , Molinelli S , Riboldi M , Baroni G . Patient‐specific validation of deformable image registration in radiation therapy: overview and caveats. Med Phys. 2018;45(10):e908‐e922. 30168155 10.1002/mp.13162

[22] Fippel M . Fast Monte Carlo dose calculation for photon beams based on the VMC electron algorithm. Med Phys. 1999;26(8):1466‐1475. 10501045 10.1118/1.598676

